# Effects of Exercise Duration and Intensity on Maximal Exercise Capacity Over 6 Months in Coronary Heart Disease and Type 2 Diabetes—A Secondary Analysis of the LeIKDTrial

**DOI:** 10.1111/sms.70209

**Published:** 2026-01-17

**Authors:** Felix Gass, Sophia M. Dinges, Isabel Fegers‐Wustrow, Felix Freigang, Patrizia Maier, Matthias Arnold, Ephraim B. Winzer, Frank Edelmann, Oliver Wolfram, Julia Brandts, Bernd Wolfarth, Marcus Dörr, Rolf Wachter, Björn Hackenberg, Sarah Rust, Thomas Nebling, Volker Amelung, Martin Halle, Stephan Mueller

**Affiliations:** ^1^ Technical University of Munich, TUM School of Medicine and Health, Department for Preventive Sports Medicine and Sports Cardiology TUM University Hospital Munich Germany; ^2^ DZHK (German Centre for Cardiovascular Research) Partner Site Munich Heart Alliance Munich Germany; ^3^ Inav—Private Institute for Applied Health Services Research gm, bH Berlin Germany; ^4^ Center for Health Services Research Brandenburg Medical School Theodor Fontane Berlin Germany; ^5^ Department for Internal Medicine and Cardiology Technische Universität Dresden, Heart Centre Dresden, University Hospital Dresden Germany; ^6^ Department of Cardiology Angiology and Intensive Care Medicine, Campus Virchow Klinikum, Deutsches Herzzentrum der Charité Berlin Germany; ^7^ Charité‐Universitätsmedizin Berlin, Corporate Member of Freie Universität Berlin and Humboldt‐Universität zu Berlin Berlin Germany; ^8^ DZHK (German Centre for Cardiovascular Research), Partner Site Berlin Berlin Germany; ^9^ Department of Cardiology and Angiology University Hospital Magdeburg Magdeburg Germany; ^10^ Department of Internal MedicineI University Hospital Aachen Aachen Germany; ^11^ Department of Sports Medicine Humboldt University and Charité University School of Medicine Berlin Germany; ^12^ Institute for Community Medicine (SHIP/KEF) University Medicine Greifswald Greifswald Germany; ^13^ DZHK (German Centre for Cardiovascular Research), Partner Site Greifswald Greifswald Germany; ^14^ Department of Cardiology University Hospital Leipzig Leipzig Germany; ^15^ Department of Cardiology and Pneumology University Medical Center Göttingen Göttingen Germany; ^16^ DZHK (German Centre for Cardiovascular Research) Partner Site Göttingen Göttingen Germany; ^17^ IDS Diagnostic Systems GmbH Oberau Germany; ^18^ Techniker Krankenkasse Hamburg Germany

**Keywords:** cardiorespiratory fitness, digital health, endurance training, exercise training, peak oxygen consumption, telemedicine

## Abstract

Exercise training is recommended in coronary heart disease (CHD) and type 2 diabetes (T2DM) patients alike; however, uncertainty remains on the influence of exercise intensity and duration in older patients with both entities. To address this, we performed a secondary analysis including 201 patients (67.9 ± 8.2 years; 84.1% men) from the LeIKD trial (NCT038359), which introduced 6 months of home‐based telemedicine‐supported exercise intervention in patients with CHD and T2DM. We assessed the relationships between exercise duration and intensity with change in peak oxygen uptake (V̇O_2_peak) (simple and multiple regression analyses, *α* = 0.05). V̇O_2_peak increased by 0.42 mL/kg/min per hour of endurance exercise/week (95% CI: 0.17–0.66, *p* = 0.001). Exercise intensity was not significantly associated with the change in V̇O_2_peak (*p* = 0.10). In a subgroup of patients with high adherence (≥ 66.7% of prescribed total duration and meeting prescribed exercise duration in ≥ 50% of weeks of intervention), a 10% increase in exercise intensity (mean % heart rate reserve (HRR)) was associated with an increase in V̇O_2_peak of 0.26 mL/kg/min (95% CI: 0.00–0.52; *p* = 0.05). Longer training duration within the initial 2 weeks of intervention was significantly associated with high adherence over 6 months (increased likelihood per 10 min/week: OR of 1.09 [95% CI: 1.05–1.14], *p* < 0.001). Therefore, exercise duration but not intensity influences changes in V̇O_2_peak during exercise intervention in a high‐risk population of older patients with CHD and T2DM. In patients with high adherence to exercise duration, higher exercise intensity led to an additional increase in V̇O_2_peak. Training duration within the first 2 weeks was an important predictor of long‐term adherence.

**Trial Registration:** https://www.clinicaltrials.gov: Identifier: NCT038359

## 1 Introduction

Exercise training is a well‐established treatment strategy to positively influence the progression of both coronary heart disease (CHD) and type 2 diabetes mellitus (T2DM) [1, 2, 3]. Yet, as with any form of treatment, adherence plays a key role in the success of exercise interventions [4]. Problematically, adherence cannot be directly observed during home‐based exercise interventions and to date, there is no widely applied gold standard measure to capture the extent to which an intervention is being used in an off‐site setting [5]. While many studies report adherence as a simple proportion of completed sessions (commonly assessed as a binary yes/no variable), measures of exercise training duration and intensity are often not reported or included in the definition of adherence [6].

Looking at current guideline recommendations, there is a distinction regarding the required exercise duration at different intensity levels, most commonly 150 min of moderate or 75 min of vigorous intensity per week [7]. However, there is no clear consensus whether intensity or total duration mostly determine the health related outcomes of exercise [8].

To determine the exercise intensity domains (e.g., light, moderate, vigorous) for exercise prescription, evaluating patients via cardiopulmonary exercise testing (CPET) is of paramount importance as it allows to measure the individual physiological responses at varying exercise intensities under consideration of the patients' health status [9]. Moreover, it allows to measure peak oxygen uptake (V̇O_2_peak)—the gold standard parameter for exercise capacity—which is also associated with CVD readmission and all‐cause mortality in cardiac patients [10]. However, since it is not feasible to continuously monitor V̇O_2_ during exercise training, different measures are required to track intensity during exercise interventions. Here, a commonly recommended measure is the percentage of heart rate reserve (HRR) [6, 9].

Given the remaining uncertainty on the influence of exercise intensity and duration on exercise capacity, especially in older high‐risk patients with cardiovascular disease, we assessed the associations between exercise duration and intensity with the change in V̇O_2_peak in patients with CHD and T2DM, using 6‐month data from the home‐based and telemedicine‐supported ‘Lifestyle Intervention in Chronic Ischaemic Heart Disease and Type 2 Diabetes’ (LeIKD) study. Furthermore, we aimed to determine factors associated with adherence to the prescribed exercise plans over 6 months.

## 2 Methods

The LeIKD study protocol, baseline characteristics as well as primary results have been published previously [11, 12, 13]. In brief, 502 patients were randomized (1:1) to a telemedicine‐supported home‐based lifestyle intervention (consisting of individualized exercise training prescriptions via a smartphone application, nutritional recommendations and health literacy training) or usual care over 12 months. Within the first 6 months, patients in the lifestyle intervention group also received regular feedback phone calls to discuss their progress within the exercise intervention. The primary endpoint was the change in HbA_1c_ after 6 months. A total of 402 participants (201 intervention, 201 usual care) completed the first 6 months. The 201 intervention group (IG) participants who completed the supervised phase and performed the 6‐month examinations are included in this sub‐analysis.

All assessments were performed according to standard operating procedures. For CPET, all sites were instructed to perform baseline and follow‐up measurements on the same bicycle ergometer and with the same metabolic cart for each individual patient. V̇O_2_peak was defined as the highest 30‐s average at the end of the incremental ramp test, analyzed centrally at the CPET core laboratory in Munich, Germany. At baseline, CPET was also used to determine specific heart rate (HR) zones to prescribe individualized exercise training for all participants in the IG. All patients received an exercise training plan via a smartphone application (IDS Diagnostic Systems AG, Ettlingen, Germany). Here, training duration and HRs were recorded, utilizing a connected HR‐sensor (Polar H7, Polar, Finland). Participants were divided into four different fitness levels based on CPET results. For endurance exercise, patients were allocated to these levels based on %‐predicted V̇O_2_peak of less than 75.0%, 75.1%–90.0%, 90.1%–110.0%, and more than 110.0%. Allocation to the four different fitness levels determined initial weekly training duration, the amount of recommended moderate and vigorous exercise, as well as progression (increase of duration and intensity) over the 6‐month period. However, independent of fitness levels, the recommendations consisted of four sessions of endurance as well as two sessions of strength‐based training per week, of which only the endurance training was included in this analysis. Prescribed endurance exercise duration increased over time (from 10 min per session up to 30–50 min per session). Session intensity varied between low, moderate, and vigorous (up to the first ventilatory threshold for low, between the first and 50% of the second ventilatory threshold for moderate, and up to the second ventilatory threshold for vigorous intensity sessions) and was further differentiated between continuous and interval training. If the second ventilatory threshold was not detectable, we used 50% of the difference between the first ventilatory threshold and V̇O_2_peak as approximation of critical power. If heart rate was not usable (e.g., due to arrhythmias), exercise intensities were prescribed using the Borg rating of perceived exertion scale.

Patients also had the possibility to perform additional sessions without prescribed intensity zones. All recorded sessions were evaluated with a customized template in Microsoft Excel and manually checked for plausibility. For the present analysis, intensities were calculated as the mean %‐HRR of all endurance exercise sessions. Further, the %‐time patients exercised within their recommended heart rate zones was also calculated. If HR measurements were classified as invalid/unreliable (e.g., very strong fluctuations), these sessions were excluded from the mean HRR calculation but still included in the average minutes and sessions of endurance training per week. In accordance with the per‐protocol analysis of the LeIKD main analysis [13], adherence to the exercise intervention was defined as having performed ≥ 66.7% of the overall recommended training duration and reaching the prescribed training duration in ≥ 50% of the weeks. However, for this secondary analysis, adherence was solely based on the ‘objective’ training recordings via the LeIKD smartphone application without considering the documentation of the regular feedback sessions.

Associations between training characteristics and change in V̇O_2_peak were evaluated via simple and multiple linear regression (including adjustment for baseline V̇O_2_peak). In the multiple linear regression models, we further included the different combinations of duration/volume [average min/week; Adherence (yes)] and intensity (mean %‐HRR; %‐recommended) parameters in a single model to evaluate their combined and adjusted effects. Associations between (pre‐selected) patient characteristics (age, sex, BMI, and baseline V̇O_2_) and the training duration within the first 2 weeks of the intervention with the 6‐month adherence were evaluated via multiple logistic regression. Variance Inflation Factors (VIF) were calculated for all variables to check for multicollinearity in the multiple regression models. All analyses were performed using R Statistical Software (Version 4.3.1; Foundation for Statistical Computing) using a significance level of *α* = 5% and no adjustment for multiple testing.

## 3 Results

The baseline and training characteristics of the included participants are shown in Tables 1 and 2. Patients (67.9 ± 8.2 years, 84.1% men) performed a mean of 78.0 ± 94.2 min of endurance training per week across 3.3 ± 3.1 sessions with a session duration of 19.2 ± 17.8 min at 51.9% ± 17.7%‐HRR. In total, patients performed 12 714 sessions of endurance exercise, of which 59% included a HR recording. Of the 201 included patients, 142 (70.6%) provided at least one HR recording, and for those, HRs were recorded in a median (IQR) of 76.9% (52.2%–94.5%) of endurance sessions. If HR was recorded, the recording on average lasted for 97% of the session duration. A total of 75 patients (37%) met the objective adherence criteria based on exercise training recordings over 6 months.

**TABLE 1:** Baseline characteristics.

Characteristic	*N* = 201
Sex	
Male	169 (84.1%)
Female	32 (15.9%)
Age at inclusion, years	67.9 (8.2)
Body mass index, kg/m^2^	30.3 (5.0)
Duration of CHD, years	8.7 (7.7)
CHD classification	
No relevant stenosis (< 50%)	38 (18.9%)
1‐vessel disease	40 (19.9%)
2‐vessel disease	42 (20.9%)
3‐vessel disease	48 (23.9%)
Left main coronary disease	4 (2.0%)
Unknown	29 (14.4%)
CCS‐score	
Grade 0	166 (83.0%)
Grade I	29 (14.5%)
Grade II	4 (2.0%)
Grade III‐IV	1 (0.5%)
Previous myocardial infarction	
Yes	68 (33.8%)
No	133 (66.2%)
Coronary revascularization	
Yes	110 (54.7%)
No	91 (45.3%)
Coronary artery bypass graft	
Yes	28 (13.9%)
No	173 (86.1%)
Duration of type 2 diabetes, years	12.6 (8.9)
HbA_1c_ %	6.8 (0.9)
Hypertension	
Yes	186 (92.5%)
No	15 (7.5%)
Hyperlipidemia	
Yes	171 (85.1%)
No	30 (14.9%)
Peak oxygen consumption	
Absolute units (L/min) [No.]	1.74 ± 0.439 [193]
Relative units (mL/kg/min) [No.]	19.1 (4.71) [193]
% predicted norm values [No.]	84.1 (17.2) [193]
Having an own mobile device	
Yes	171 (89.1%)
No	21 (10.9%)

*Note:* Values are shown as *n* (%) or mean (SD).

**TABLE 2:** Training characteristics.

Characteristic	Mean ± SD	Median (IQR)	Minimum/Maximum
Duration (min/week)	78.0 ± 94.2	57.0 (0.5 to 115.2)	0/569.9
Frequency (sessions/week)	3.3 ± 3.1	2.7 (0.1 to 5.8)	0/19.2
Duration per session (min/session)	19.2 ± 17.8	15.9 (13.5 to 27.3)	2.7/98.4
Intensity (%‐HRR)	51.9 ± 17.7	51.9 (40.1 to 61.5)	8.8/101.5

Abbreviations: HRR, heart rate reserve; min, minutes; V̇O_2_, oxygen uptake.

**FIGURE 1:**
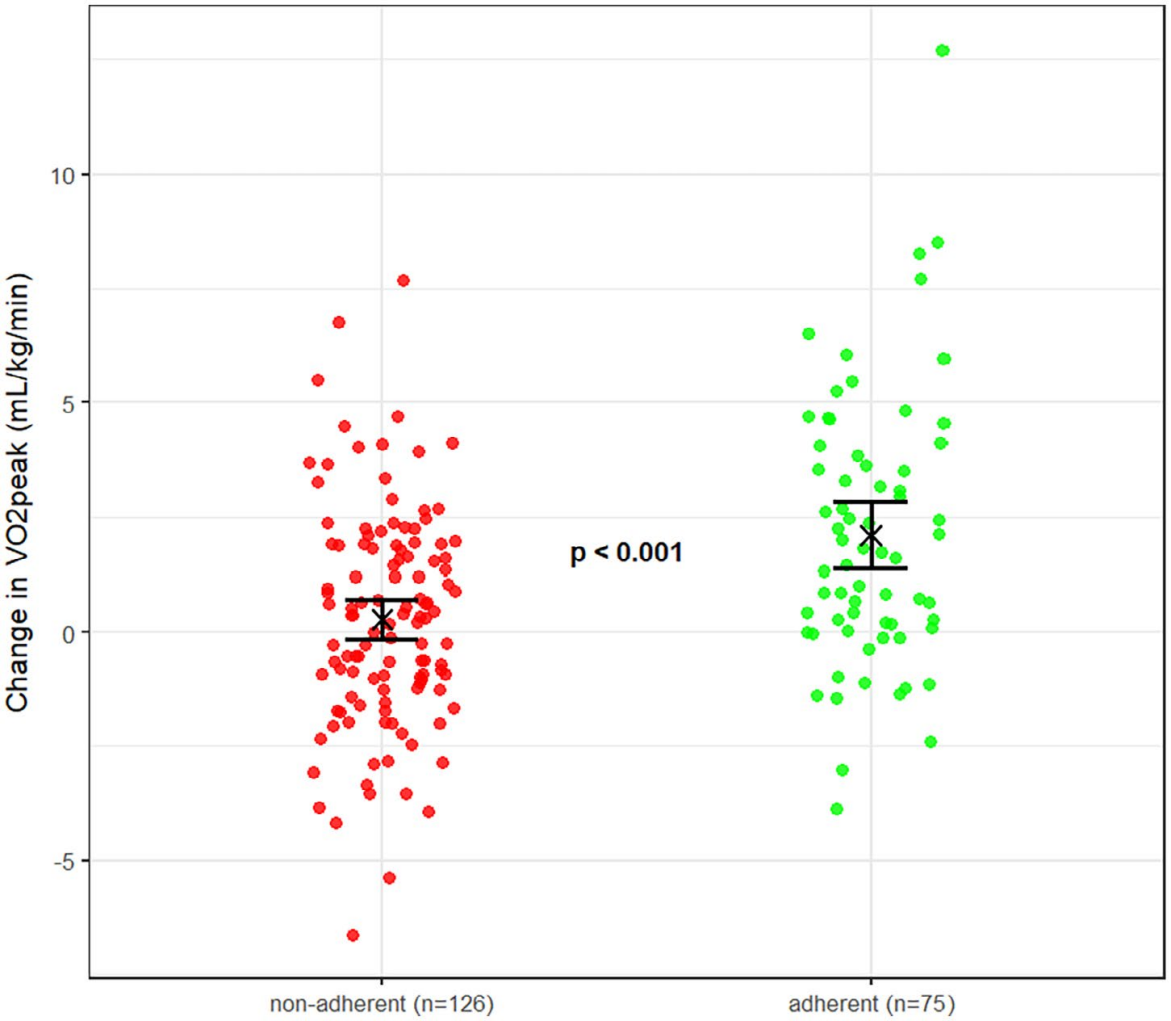
Change in peak oxygen consumption compared between patients classified as adherent to the exercise intervention and non‐adherent patients. The R function ‘jitter’ has been used to reduce overplotting. Adherence was defined as having performed ≥ 66.7% of the overall recommended training duration and reaching the prescribed training duration in ≥ 50% of the weeks, based on the training recordings of the LeIKD smartphone application without considering additional documentation.

CPET data at both baseline and 6‐month follow‐up were available for 184 patients. Among those, V̇O_2_peak changed from 19.1 ± 4.8 mL/kg/min (1.74 ± 0.440 L/min) at baseline to 20.1 ± 5.4 mL/kg/min (1.78 ± 0.475 L/min) after 6 months. Comparing the effects between the participants classified as adherent (1.95 ± 2.94 mL/kg/min) versus non‐adherent (0.25 ± 2.39 mL/kg/min), the change in V̇O_2_peak was on average 1.70 mL/kg/min ([95% CI: 0.93 to 2.48], *p* < 0.001) higher if participants adhered to the intervention programme (see Figure 1).

**FIGURE 2:**
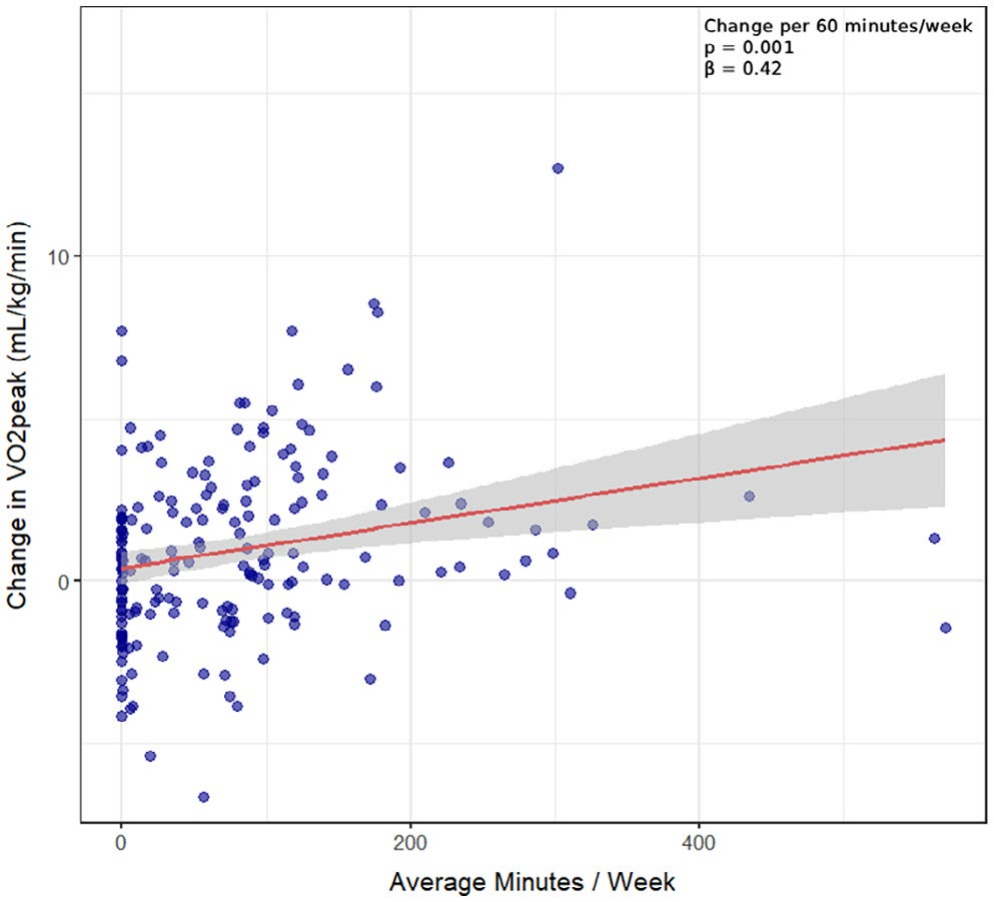
Association of minutes of recorded endurance exercise with change in peak oxygen consumption between baseline and the 6‐month re‐examination. The red line represents the linear regression model showing the association between average exercise duration per week (in minutes) and the change in V̇O_2_peak (mL/kg/min) between baseline and the 6‐month re‐examination. The shaded gray area indicates the 95% confidence interval of the regression slope. The dark blue points depict individual participants' data, with variations in color intensity due to overlapping points (transparency has been applied to reduce overplotting). Participants with 0 min of recorded exercise: *N* = 46.

The results of the simple regression models are presented in Table 3. Regarding the metrics of weekly training duration and sessions per week, both significantly improved the change in V̇O_2_peak (by 0.42 mL/kg/min [95% CI: 0.17 to 0.66] for every additional hour, *p* = 0.001 (see Figure 2), and 0.26 mL/kg/min [95% CI: 0.14 to 0.38] for every additional session, *p* < 0.001). Intensity, as measured by average %‐HRR, failed to reach statistical significance (*p* = 0.10). On the other hand, when evaluated as %‐time within the prescribed training prescription zones, every 10% of exercise time within the recommended HR zones was associated with a 0.17 mL/kg/min ([95% CI: 0.02 to 0.33], *p* = 0.03) higher change in V̇O_2_peak. Adjusting for baseline V̇O_2_peak did not significantly alter the observed results (Table 3).

**TABLE 3:** Univariate linear regression models for the associations between exercise training characteristics and change in V̇O_2_peak (mL/kg/min).

Independent variable	*N*	Change in V̇O_2_peak (ß‐coefficient)	95% CI lower to upper	*p* value	*R* ^2^
Unadjusted linear regression models					
Adherence (yes)	184	1.70	0.93 to 2.48	< 0.001	0.09
Average min/week, per 60 min	184	0.42	0.17 to 0.66	0.001	0.06
Average sessions/week, per session	184	0.26	0.14 to 0.38	< 0.001	0.09
Mean %‐HRR, per 10%	127	0.22	−0.05 to 0.49	0.10	0.02
%‐recommended, per 10%	184	0.17	0.02 to 0.33	0.03	0.03
Adjusted for baseline V̇O_2_peak					
Adherence (yes)	184	1.74	0.96 to 2.52	< 0.001	0.09
Average min/week, per 60 min	184	0.46	0.21 to 0.72	< 0.001	0.06
Average sessions/week, per session	184	0.28	0.15 to 0.40	< 0.001	0.09
Mean %‐HRR, per 10%	127	0.22	−0.05 to 0.49	0.11	0.01
%‐recommended, per 10%	184	0.20	0.04 to 0.36	0.02	0.02

Abbreviations: %, recommended, percentage of time in recommended heart rate zone; HR, heart rate; HRR, heart rate reserve; min, minutes; V̇O_2_, oxygen uptake.

The results of the multiple linear regression models are shown in Table 4. In a model including both weekly training duration as well as training intensity, neither was significantly associated with the change in V̇O_2_peak (*p* = 0.06 and *p* = 0.09). When including adherence as well as %‐HRR, both were significantly associated with the change in V̇O_2_peak (by 1.56 mL/kg/min [95% CI: 0.64 to 2.49] for adherent participants, *p* = 0.001, and 0.26 [95% CI: 0.00 to 0.52] per 10% higher %‐HRR, *p* = 0.05). When including the variables of training duration (per session) as well as sessions per week (frequency), V̇O_2_peak increased by 0.27 mL/kg/min ([95% CI: 0.14 to 0.40], *p* < 0.001) per additional weekly exercise session, whereas training duration per session was not significantly associated (*p* = 0.69) with the change in V̇O_2_peak. The %‐time that participants exercised within their recommended HR zones was not significantly associated with the change in V̇O_2_peak in any of the investigated multiple regression models. Further adjustment for age and sex did not significantly change the results (data not shown).

**TABLE 4:** Multiple linear regression models for the associations between exercise training characteristics and change in V̇O_2_peak (mL/kg/min).

Model	*N*	Independent variables	Change in V̇O_2_peak (ß‐coefficient)	95% Ci lower to upper	*p*	VIF	Adjusted *R* ^2^	Model *p* value
1.	127	Average min/week, per 60 min Mean %‐HRR, per 10% Baseline V̇O_2_peak (mL/kg/min)	0.31 0.23 −0.04	−0.02 to 0.64 −0.04 to 0.50 −0.14 to 0.06	0.06 0.09 0.44	1.09 1.01 1.09	0.03	0.10
2.	184	Average min/week, per 60 min %‐ recommended, per 10% Baseline V̇O_2_peak (mL/kg/min)	0.42 0.16 −0.07	0.17 to 0.66 −0.02 to 0.31 −0.16 to 0.01	0.001 0.06 0.08	1.11 1.11 1.15	0.07	< 0.01
3.	127	Adherence (yes) Mean %‐HRR, per 10% Baseline V̇O_2_peak (mL/kg/min)	1.56 0.26 −0.03	0.64 to 2.49 0.00 to 0.52 −0.13 to 0.07	0.001 0.05 0.54	1.02 1.01 1.01	0.08	< 0.01
4.	184	Adherence (yes) %‐ recommended, per 10% Baseline V̇O_2_peak (mL/kg/min)	1.58 0.11 −0.05	0.77 to 2.40 −0.05 to 0.27 −0.13 to 0.03	0.001 0.19 0.25	1.10 1.18 1.08	0.09	< 0.01
5.	179	Average min/session, per 10 min Average sessions/week Baseline V̇O_2_peak (mL/kg/min)	0.04 0.27 −0.06	−0.18 to 0.28 0.14 to 0.40 −0.15 to 0.02	0.69 < 0.001 0.16	1.11 1.12 1.08	0.08	< 0.01

Abbreviations: %, recommended, percentage of time in recommended heart rate zone; HR, heart rate; HRR, heart rate reserve; min, minutes; R^2^, coefficient of determination; VIF, variance inflation factor; V̇O_2_, oxygen uptake.

**FIGURE 3:**
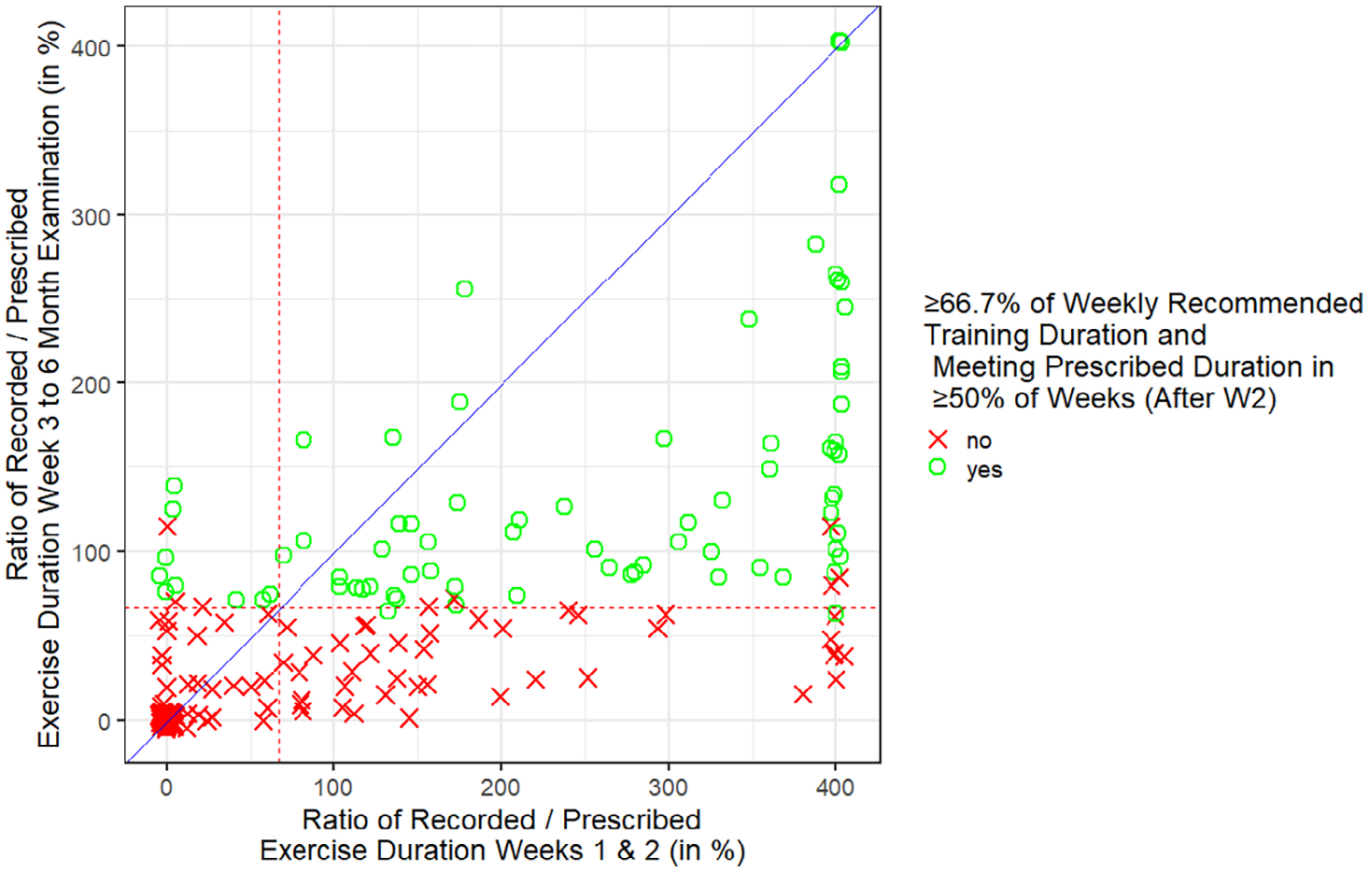
Association between the ratio of recorded versus prescribed training duration within the first two training weeks and between the third training week 3 and the 6‐month examination. The red‐dashed lines indicate the pre‐defined adherence cutoff of the main study (66.7%). Fulfillment of the second adherence criteria between week 3 and the 6‐month evaluation (reaching the prescribed training duration in ≥ 50% of the intervention weeks) is depicted via green circles (yes) or red crosses (no). The blue line shows the line of equality between the x‐ and the y‐axis. The R function ‘jitter’ has been used to reduce overplotting. Furthermore, outliers > 400% were plotted as 401% (*n* = 31).

Table 5 shows the results of the logistic regression model to evaluate predictors of adherence after 6 months of exercise intervention. Only the training duration within the first 2 weeks of the intervention was significantly associated with the likelihood of being classified as adherent after 6 months. Each additional 10 min of exercise per week in the first 2 weeks was associated with increased odds (OR of 1.09 [95% CI: 1.05 to 1.14], *p* < 0.001) of being adherent at 6 months. Figure 3 shows the association between the ratio of recorded and prescribed weekly exercise duration within the first 2 training weeks and the adherence between the 3rd training week and the 6‐month examination. Of 92 patients with an initially low training duration (< 66.7% of prescribed duration), only 9 (9.8%) were classified as adherent between week 3 and the 6‐month evaluation. Conversely, this was the case for 66 of 109 patients (60.6%) with an initial training duration ≥ 66.7% of the prescribed duration.

**TABLE 5:** Multiple logistic regression results for the likelihood of being classified as adherent towards the intervention (*n* = 185).

Independent variables	Odds ratio [95% CI]	*p*	95% CI lower to upper	VIF
Initial training duration, per 10 min/week	1.09	< 0.001	1.05–1.14	1.11
Baseline V̇O_2_peak	1.01	0.87	0.92–1.09	1.48
BMI	1.02	0.54	0.95–1.11	1.36
Sex (male)	1.73	0.30	0.64–5.26	1.05
Age	1.01	0.54	0.97–1.06	1.10

Abbreviations: BMI, body mass index; initial training duration, average minutes of endurance training per week in weeks 1 + 2; min, minutes; V̇O_2_, oxygen uptake; VIF, variance inflation factor.

## 4 Discussion

In this secondary analysis of the home‐based, telemedicine‐supported LeIKD trial, the major findings are: (1) Mean duration of endurance training per week was significantly associated with the change in V̇O_2_peak; (2) Sessions frequency, but not session duration, was significantly associated with the change in V̇O_2_peak; (3) Exercise intensity was not significantly associated with the change in V̇O_2_peak in simple linear regression analysis, but was associated with an additional increase in V̇O_2_peak in those who closely adhered to the prescribed exercise duration; (4) Training duration within the first 2 weeks was significantly associated with the likelihood of being adherent over 6 months.

Overall, patients performed a mean of 78.0 (±94.2) minutes of endurance training per week at a mean intensity of 51.9 (±17.7) %‐HRR after 6 months. Adherence to the exercise intervention as defined by the study protocol (having performed ≥ 66.7% of the recommended training duration and reaching the prescribed training duration in ≥ 50% of the weeks) was strongly associated with the change in V̇O_2_peak after 6 months with an average 1.70 mL/kg/min (95% CI: 0.93–2.48), *p* < 0.001 higher change in V̇O_2_peak in adherent compared to non‐adherent patients. This adds to the findings in the primary publications' per‐protocol analysis, where patients classified as adherent towards both the exercise and nutrition intervention showed a higher reduction in the studies' primary endpoint (glycated hemoglobin) when compared to non‐adherent patients [13]. Overall, only 40% of patients were defined as adherent to the exercise intervention. Compared to centre‐based interventions, low adherence is a common problem in home‐based exercise interventions where adherence towards the programme can be as low as 30% [14]. Yet, the adherence was lower than expected, especially given the utilization of telemedicine‐supported patient supervision, which was developed incorporating feedback of a cardiovascular patient population, a feature generally expected to increase adherence in home‐based interventions [15]. This shows that when designing a telemedicine‐supported supervision concept, it is not only important to address patient needs, but also to consider their motivation and willingness to work with a novel and unfamiliar treatment strategy. This leaves a lot of room to improve adherence in future exercise trials by expanding on the telemedical concept—and especially further adapting it to older and multimorbid patient populations [5].

The strong association between weekly training duration and change in exercise capacity is in line with the findings of previous studies, which showed that higher weekly training duration is significantly associated with a larger improvement in V̇O_2_peak [16, 17, 18, 19, 20, 21, 22, 23, 24]. Splitting weekly exercise duration into its determinants (i.e., session frequency × session duration), session frequency appeared to be more important. In simple linear regression, training frequency explained 9% of variance in change in V̇O_2_peak—a significant result, given that individual characteristics (such as sex, age or BMI) can account for 15%–21% and heritable factors for up to 50% of the observed variation in the change of V̇O_2_peak after exercise training [25]. Furthermore, in a regression model including the mean frequency as well as duration of exercise sessions, only the frequency was significantly associated with the change in V̇O_2_peak, with every additional weekly session being associated with a 0.25 mL/kg/min higher change in V̇O_2_peak. This suggests that in a multimorbid patient population of CHD and T2DM, motivating participants to engage in shorter and more frequent exercise sessions may be more promising as compared to less regular and longer sessions. This adds evidence to the ongoing discussion that training frequency might be more important than bout‐duration when it comes to the observed changes in cardiometabolic health—where every minute of exercise matters and shorter bouts might be easier to perform and could therefore lead to an increased adherence in cardiac patients [26, 27].

Regarding intensity, some studies in patients with CVD show intensity to be positively associated with the change in cardiorespiratory fitness, while others found that exercising at lower intensity levels is already sufficient or potentially even superior to improve V̇O_2_peak [19, 28, 29]. While not significantly associated with the change in V̇O_2_peak in the simple regression analyses or when adjusted to weekly duration, higher mean %‐HRR was significantly associated with the change in V̇O_2_peak when combined with the binary adherence variable (yes/no). In summary, surpassing a specific minimum training duration (preferably with a high session frequency) seems to be the initial and more important factor determining the observed changes in V̇O_2_peak—but in adherent patients, training intensity may be a further differentiator.

Another crucial finding is the importance of initial training duration. Multiple logistic regression identified only the initial training duration in the first 2 weeks of the intervention as a significant predictor of the 6‐month adherence. Plotting the ratio of recorded and prescribed weekly exercise duration in the first two training weeks against the adherence between the 3rd training week and the 6‐month examination highlights the main problem of the LeIKD study: to initiate regular exercise training in predominantly sedentary, multimorbid and overweight patients with CHD and T2DM. Indeed, only 9 of 92 patients (9.8%) with initially short training duration were classified as adherent between week 3 and the 6‐month evaluation, whereas 66 of 109 patients (60.6%) with initially long training duration met these adherence criteria.

Consequently, initiating all patients into the intervention, not maintenance of initially adherent patients, posed the major problem in the LeIKD study. Future research is needed on how to reduce the barriers of entry into regular home‐based exercise training—especially for cardiac patients, who in many cases have no previous experience regarding structured physical exercise and are often scared of how the intervention might affect their heart condition.

## 5 Limitations

This secondary analysis of the LeIKD trial has several limitations and should be considered exploratory, given there was no adjustment for multiple testing. First, part of the intervention occurred during the COVID‐19 pandemic, which might have impacted the observed adherence towards the exercise programme. Second, the telemedical concept initially suffered from slight technical problems (specifically compatibility issues with certain smartphone brands), which were mostly solved over the course of the study but might have impacted the adherence of participants who were recruited early in the trial. Third, patients may have performed additional exercise sessions without recording them, which could have impacted the observed outcomes. Lastly, 41% of all sessions had no HR recordings and approximately 1/3 of patients did not record any HRs. Therefore, the intensity of these sessions remains unknown, and the analyses involving the intensity variable are based on a smaller sample size and statistical power. Of 142 (70.6%) patients that provided at least one HR recording, HRs were recorded in a median (IQR) of 76.9% (52.2% to 94.5%) of endurance sessions, which further emphasizes the significant individual variations. Wearing a chest‐strap HR sensor and repeatedly connecting it with their mobile device proved to be uncomfortable and overly complex for many patients, which has been reported as a problem of these devices in elderly and especially overweight populations [22]. Therefore, future studies with more complete HR information are needed to more precisely assess the impact of intensity on the change of V̇O_2_peak. Finally, this sub‐analysis of the LeIKD included only endurance exercise data and it is therefore not possible to draw any conclusions regarding the impact of the strength exercise session, which might also have impacted the observed results.

## 6 Perspective

In a high‐risk patient population suffering from both CHD and T2DM, change in V̇O_2_peak was primarily related to weekly exercise duration and frequency, but not intensity. However, in a subgroup of patients with high adherence to the prescribed exercise duration, higher exercise intensity led to an additional increase in V̇O_2_peak. Therefore, it appears to be of paramount importance to engage multimorbid patient populations in regular endurance exercise by focusing on the weekly training duration and frequency rather than training intensity. For home‐based telemedicine‐supported exercise interventions, early engagement seems to be very important since training duration within the first two intervention weeks emerged as a key predictor of longer‐term training adherence.

## Funding

This work was supported by the Federal Joint Committee (Innovationsfonds des Gemeinsamen Bundesausschusses; reference 01NVF17015 (to B.H., V.A. and M.H.)). The funder had no role in study design, data collection and analysis, decision to publish, or preparation of the manuscript.

## Ethics Statement

The study protocol of the main study was approved by the ethics committee of the Technical University of Munich on 7 May 2018 (reference: 144/18 S) and the local ethics committees at the Universities of Berlin, Aachen, Magdeburg, Dresden, Leipzig, Greifswald and Freiburg, and the ethics committees of the Medical Associations of Hesse and Baden‐Württemberg. A revised trial protocol (including a refinement of the inclusion criterion for the diagnosis of T2DM, that is, ‘HbA1c ≥ 6.5% or antidiabetic medication at the time of on‐site screening’ in addition to ‘ICD‐10: E11’) had also been approved by the ethics committees of the Technical University of Munich (reference: 144/18 S‐AS) and the participating sites.

## Consent

All participants provided written informed consent.

## Conflicts of Interest

S.M. reported personal fees from Bristol‐Myers Squibb (consulting services) outside the submitted work. E.B.W. reported grants from Boehringer Ingelheim and personal fees from Amarin, Amgen, AstraZeneca, Daiichi Sankyo, Bayer, Boehringer Ingelheim, CVRx and Novartis outside the submitted work. F.E. reported personal fees from AstraZeneca, Bayer, Berlin Chemie, Boehringer Ingelheim, CVRx, Medtronic, Merck, MSD, Novartis, Pfizer, PharmaCosmos, Resmed, Servier and Vifor Pharma; nonfinancial support from Novartis and grants from AstraZeneca, Boehringer Ingelheim, Servier and Thermo Fischer Scientific outside the submitted work. R.W. reported personal fees from AstraZeneca, Bayer, BMS, Boehringer Ingelheim, CVRx, Daiichi, Medtronic, Novartis, Pfizer, Pharmacosmos and Servier and research support from Boehringer Ingelheim, Bundesministerium für Bildung und Forschung, Deutsche Forschungsgemeinschaft and European Union and Medtronic outside the submitted work. V.A. reported personal fees from MSD, Berlin Chemie, Janssen, Coloplast and Novo Nordisk (consulting fees and honoraria for lectures) and is the president of the German Managed Care Association (since 2007) outside the submitted work. M.H. reported honoraria for lectures from Abbott, Amgen, AstraZeneca, Boehringer Ingelheim, Bristol‐Myers Squibb, Daiichi Sankyo, Lilly, Medi, Novartis, Pfizer and Roche; consulting fees from Medical Park and is the past president of the European Association of Preventive Cardiology (presidency 2020–2022) outside the submitted work. J.B. received speaker honoraria from Amgen, Daiichi Sankyo, Lilly Eli, Novartis, Novo Nordisk and Sanofi. All other authors declare no conflicts of interest.

## Data Availability

As patients have not explicitly consented to sharing pseudonymized data, we are not allowed to share the individual participant data for legal reasons.
